# Fungicide sensitivity levels in the Lithuanian *Zymoseptoria tritici* population in 2021

**DOI:** 10.3389/fpls.2022.1075038

**Published:** 2023-01-11

**Authors:** Karolina Lavrukaitė, Thies M. Heick, Jūratė Ramanauskienė, Rita Armonienė, Antanas Ronis

**Affiliations:** ^1^ Lithuanian Research Centre for Agriculture and Forestry, Institute of Agriculture, Akademija, Lithuania; ^2^ Department of Agroecology, Aarhus University, Slagelse, Denmark

**Keywords:** fungicide resistance, DMI, QoI, QiI, SDHI, Septoria tritici blotch

## Abstract

*Zymoseptoria tritici* causes the disease known as septoria leaf blotch in winter wheat and is a major factor in yield loss worldwide. Farmers are inclined to use fungicides to protect their crops; however, the efficacy of these measures is rapidly decreasing due to the natural mechanisms of mutation emergence in pathogen populations. Increasing fungicide resistance is being recorded worldwide, therefore, screening of the current situation in Lithuania is essential to determine the subsequent steps of crop protection strategies. In this study, *in vitro* fungicide sensitivity tests, mutation detection, and field experiments were carried out. The mean EC_50_ values for prothioconazole-desthio and mefentrifluconazole were 0.14 and 0.28 mg/l, respectively. Increased frequency of the mutation S524T, linked to DMIs resistance, was observed. Results revealed that the dominant point mutation in the gene *CYP51* was I381V, and the most frequent *CYP51* haplotype was D13 (V136C, I381V, Y461H, S524T). The mutation G143A, linked to QoI resistance, was detected in ¾ of the population. Mutations conferring resistance to SDHIs were not detected in single pycnidium isolates. Two-year field experiments likewise showed no decline in field efficacy of SDHI fungicide in Lithuania. Moreover, the baseline sensitivity of the Lithuanian *Z. tritici* population to QiI fungicide fenpicoxamid was established. The findings of this study provide an update on the current status of fungicide resistance in the Lithuanian *Z. tritici* population.

## 1 Introduction

The estimated wheat yield loss due to pests and diseases ranges from 10.1 to 28.1% globally and reaches almost 25% in Northwest Europe (Savary et al., 2019). Despite the availability of different disease control methods, such as varietal resistance or crop rotation, continuous fungicide application is the most prevalent way of reducing yield loss (Lynch et al., 2017). However, farmers are currently facing the challenge of increased fungicide resistance in various plant pathogens (Jørgensen et al., 2018a). Over the last decade, numerous studies have reported cases of fungicide resistance in such pathogens, especially *Zymoseptoria tritici* which causes the foliar wheat disease Septoria tritici blotch (STB) (Rehfus et al., 2018; Mäe et al., 2020; Jørgensen et al., 2021a). STB is distributed worldwide and occurs in areas with moderate temperatures and higher humidity (Torriani et al., 2015). The rapid development of resistance to various classes of fungicide in *Z. tritici* had been linked to its mixed (asexual and sexual) reproductive system (Brunner et al., 2008).

Fungicides with different modes of action (MoA) are used in STB disease control, for example, 14-demethylation inhibitors (DMI), succinate dehydrogenase inhibitors (SDHI), quinone outside inhibitors (QoI), and multi-site inhibitors, such as folpet and sulfur. The use of single-site fungicides, such as DMIs, SDHIs, and QoIs, increases the risk of resistance development. The multi-site inhibitors, however, affect multiple target sites in the pathogen and, thus, the pathogen is less likely to develop resistance against it (Hobbelen et al., 2011). Nevertheless, there are currently no products containing multi-site inhibitors against *Z. tritici* in Lithuania.

QoI fungicides inhibit cellular respiration by binding to the Qo site of the cytochrome b (C*ytb*) complex. The point mutation G143A in the *Cytb* gene had been linked to resistance to QoI fungicides in *Z. tritici* (Grasso et al., 2006), and is broadly spread across Europe (Cheval et al., 2017; Heick et al., 2017; Mäe et al., 2020). In addition, resistance to QoI fungicides had been recorded to spread independently in separate locations (Torriani et al., 2009). Therefore, these fungicides are not recommended for use against STB. However, QoIs are still used in Lithuania and other Northeast European countries, mainly targeting rust diseases (Mäe et al., 2020). Following the recent introduction of a new active ingredient fenpicoxamid, another group of fungicides, which act upon a different site of *Cytb*, had been receiving more attention. This active ingredient belongs to the quinone inside inhibitors (QiI) group, which inhibit the mitochondrial respiratory bc1 complex at the Q_i_ binding site (Owen et al., 2017).

SDHIs inhibit fungal respiration by disrupting the functioning of the succinate dehydrogenase (SDH) enzyme in the pathogens’ mitochondria. The mutations conferring resistance to SDHIs occur in SDH enzyme subunits B, C, and D (Sierotzki and Scalliet, 2013). Although studies had shown good efficacy of SDHIs against *Z. tritici* (Rehfus et al., 2018; Kiiker et al., 2021), over the last two decades, a decline in sensitivity has been observed in Germany (Birr et al., 2021). Several mutations had been linked to reduced sensitivity to SDHI fungicides, such as B-T268I, C-T79N, V-N86S, and C-H152R (Rehfus et al., 2018; FRAC, 2022).

DMI fungicides target the sterol 14-α-demethylase of the pathogen, thus preventing the demethylation and production of sterols, which are essential to the maintenance of membrane fluidity and permeability (Jefcoate, 1978). The control of fungal diseases had relied heavily on DMI fungicides since their introduction to the present day (Russell, 2005; Jørgensen and Heick, 2021b; Klink et al., 2021). This fungicide group possesses not only protective, but also curative activity when used against *Z. tritici* (Jørgensen et al., 2018b). However, the reduction in *Z. tritici* sensitivity towards DMI fungicides had been recorded by several researchers worldwide (Sykes et al., 2018; Mäe et al., 2020; Jørgensen et al., 2021a; Klink et al., 2021). Declined DMI sensitivity in *Z. tritici* had been linked to multiple mechanisms, such as mutations in the target gene (*CYP51*), overexpression of the *CYP51* gene, and enhanced active fungicide efflux (Ziogas and Malandrakis, 2015). Moreover, cross-resistance within the group of DMIs adds to the reduction in sensitivity of *Z. tritici* (Omrane et al., 2017). As described previously, mutations in the *CYP51* gene are associated with a decrease in sensitivity (Jørgensen et al., 2021a). These mutations include D134G, V136A/C/G, A379G, I381V, S524T, changes among amino acids 459 to 461, and so forth (Cools and Fraaije, 2013). Various attempts have been made to classify CYP51 haplotypes. One method employed the distinction of haplotypes based on DMI resistance, whereas another evaluated the number of key alterations within a haplotype (Leroux et al., 2007; Huf et al., 2018).

The prevalence of fungicide resistance in *Z. tritici* challenges farmers and researchers to pursue novel and efficient methods of plant protection. It is essential to conduct routine monitoring in order to select the most appropriate protection strategies and fungicides for a specific region (Kiiker et al., 2021). Notably, a pattern of gradually increasing numbers of fungicide resistance cases and mutation frequencies had been observed from East to West Europe (Jørgensen et al., 2018b; Hellin et al., 2021). Thus, the main aim of this work was to determine the current status of resistance to fungicides with different MoA in relation to resistance-linked mutations in the Lithuanian *Z. tritici* population. In order to provide a comprehensive overview and a sound recommendation for farmers, a molecular mutation screening was carried out as well as *in vitro* sensitivity assays and field experiments.

## 2 Materials and methods

### 2.1 Field sampling and isolate collection

**Figure 1:**
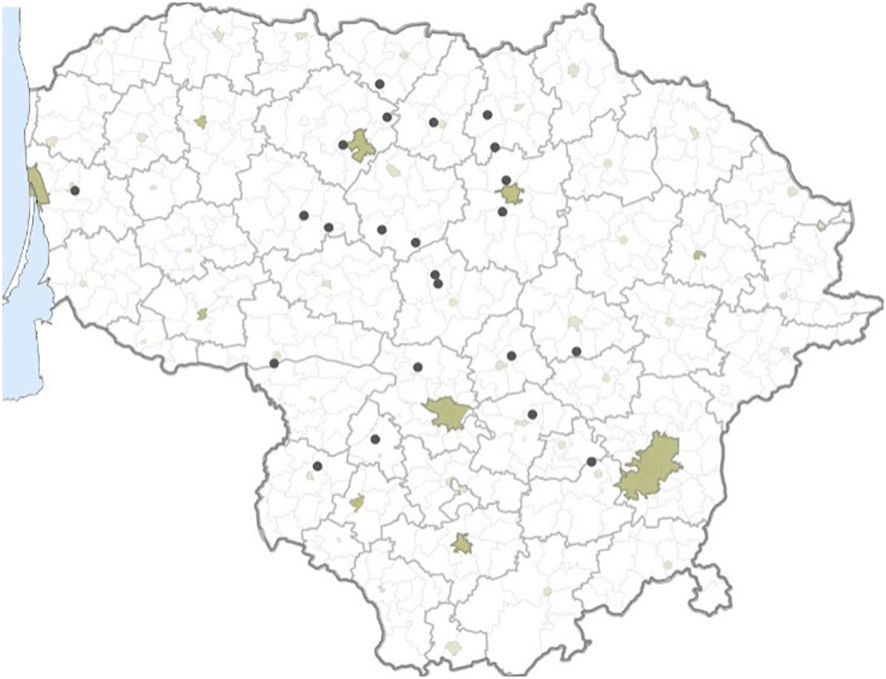
Map indicating sampling locations of the *Z. tritici* population across Lithuania in 2021.

Leaves with typical STB lesions were collected in 2021 from commercial winter wheat fields in 23 locations across Lithuania (**Figure 1**). Samples from these locations were used for *Z. tritici* single pycnidium isolates isolation and as bulk samples for DNA extraction from leaves. A total of 42 *Z. tritici* single pycnidium isolates were isolated (1-2 isolates from each of the 23 locations). Without prior surface sterilization, the 3-4 cm leaf pieces with characteristic STB lesions were placed in ø 9 cm Petri dishes, containing filter paper moistened with demineralized water, and kept at room temperature for 24 h. Using a sterile needle, cirrhi from a single pycnidium were transferred onto potato dextrose agar (PDA), containing 0.01% streptomycin. The plates containing PDA were incubated at 17°C with a 12 h white light/12 h darkness cycle for 5 days. Individual spore colonies were collected and conserved in 20% glycerol at −80°C.

### 2.2 Fungicide sensitivity test

*In vitro* sensitivity test was performed with the fungicides prothioconazole-desthio, mefentrifluconazole, and fenpicoxamid (all Sigma-Aldrich, St. Louis, MO, USA). Sensitivity tests were conducted as described by Heick et al. (2020), with the concentrations of prothioconazole-desthio set to 0.0165, 0.049, 0.15, 0.44, 1.33, 4.0, 12.0 mg L^-1^ and mefentrifluconazole and fenpicoxamid both set to 0.01, 0.02, 0.1, 0.2, 1.0, 2.0, 6.0 mg L^-1^. Sensitivity to fungicides was calculated by non-linear regression (curve-fit) as the concentration of fungicide, which inhibits fungal growth by 50% (EC_50_). The calculations were carried out using GraphPad Prism (GraphPad Software, La Jolla, CA, USA). The isolate IPO 323 (Dutch field strain first isolated in 1984) was included in the sensitivity test as a reference. Resistance factors (R.F.) were calculated using the formula: (EC_50_ of the isolate)/(EC_50_ of reference isolate IPO 323).

### 2.3 DNA extraction from a bulk of leaf samples and *Z. tritici* fungal isolates

From each sample (from 23 locations), 50 leaf cores (5 cores from 10 leaves each) containing STB lesions, were excised using a 2 mm biopsy puncher. Each bulk of samples comprised of 50 leaf cores was freeze-dried and homogenized using a Geno/Grinder^®^ 2010 (Spex^®^SamplePrep, Stanmore, United Kingdom) for 3x45 s at 1,500 rpm in 1.5 mL tubes with a steel ball (ø 5 mm) inside. The bulked samples represent the same field, from which the respective single isolates were collected. The same procedure was applied for the homogenization of fungal isolates. For each of the 42 isolates, 30 mg of fungal material were used. DNA was extracted from the homogenized leaf/fungal material using Sbeadex™ mini plant kit (LG Genomics, Teddington, UK) according to the manufacturer’s protocol on a KingFisher™ Flex Purification System (Thermo Fisher Scientific, Roskilde, Denmark) and eluted in 50 µl elution buffer

### 2.4 Molecular analysis of target site mutations

Forty-two single pycnidium isolates were tested for mutations conferring resistance to QoI, SDHI, and DMI fungicides. The presence of mutation G143A in the *Cytb* gene was determined using Kompetitive Allele Specific PCR (KASP) (LGC Genomics, Teddington, U.K.) genotyping (Kildea et al., 2014). The frequencies of mutations C-T79N and C-N86S in SDH-C genes were determined using qPCR assays as described by Hellin et al. (2021). Moreover, the DNA of bulk leaf samples from each of the 23 locations were also screened to determine the frequencies of mutations C-T79N and C-N86S in SDH-C genes.

The presence of target site mutations in the *CYP51* gene was determined, as described by Kildea et al. (2019). The complete CYP51 gene sequence in the isolates was determined by following the amplification of the gene in each isolate using three overlapping PCRs. All PCR reactions were performed using: 1 unit GoTaqFlexi DNA polymerase (Promega, Madison, USA), 5.0 μl 5× GoTaqFlexi PCR Buffer (Promega, Madison, USA), 1.5 μL MgCl_2_, 2.0 μL of dNTPs (2,5 mM each), 2,5 µl forward and reverse primers (both 10µM) (see **Table S1**), and 1.0 μL DNA and brought to a final reaction volume of 25 μL with Gibco water. The conditions for PCR reactions were as follows: initial denaturation at 94°C for 2 min, 35 cycles at 94°C for 30 s, annealing at 58°C for 30 s, and 72°C for 1 min, with the final extension step at 72°C for 10 min. PCR products were purified and sequenced by Macrogen Europe B.V. (Amsterdam, Netherlands). Sequence assembly and alignment were performed using the CLC workbench (QIAGEN, Aarhus, Denmark), where *Z. tritici CYP51* protein (GenBank accession no. AY730587) was used as a reference. According to the combination of target site mutations in the *CYP51* gene, the isolates were assigned to *CYP51* haplotypes, as described by Huf et al. (2018). All reactions described above were carried out using an Applied Biosystems ViiaTM 7 Real-time PCR system machine (Thermo Fisher Scientific, Denmark).

### 2.5 Overexpression of the *CYP51* and *MFS1* genes

The presence of insertions in the *CYP51* promoter region conferring overexpression of the gene was determined for all isolates as described by Cools et al. (2012). PCR reactions were carried out in a total volume of 25 μl containing 10.375 μl Gibco water, 5.0 μl 5× GoTaqFlexi PCR buffer (Promega, Madison, USA), 1.5 μL MgCl_2_, 2.0 μL of each dNTP, 10 μM forward primer Mg51-proF and reverse primer Mg51-seqR each, 1 unit GoTaqFlexi DNA polymerase (Promega, Madison, USA), and 1.0 μl DNA (approximately 5 ng μl^–1^). The conditions for PCR reactions were as follows: initial denaturation at 94°C for 2 min, 40 cycles at 94°C for 30 s, annealing at 60°C for 30 s, and 72°C for 1 min, with the final extension step at 72°C for 5 min. PCR reactions were carried out in an Applied Biosystems 2720 Thermal Cycler (Thermo Fisher Scientific, Denmark). The DNA amplicons were loaded on a 1% agarose gel containing SYBR^®^ Safe DNA Gel Stain (Thermo Fisher Scientific, Denmark) and run at 100 V for 45 min.

The presence of enhanced efflux, also referred to as multi-drug resistance (MDR), in the isolates was determined using PCR with specific primers MFS_2F and MFS_4R as described by Omrane et al. (2015). PCR reactions were carried out in an Applied Biosystems 2720 Thermal Cycler (Thermo Fisher Scientific, Denmark). The DNA amplicons were loaded on a 1% agarose gel containing SYBR^®^ Safe DNA Gel Stain (Thermo Fisher Scientific, Denmark) and run at 100 V for 45 min.

### 2.6 Field trials

Two field experiments were conducted on winter wheat in Lithuania to test the efficacy of fungicides with different MoA against STB in 2020 and 2021 (GPS coordinates: 55.3820, 23.8556). The experiments were set up with four replicates and complete randomization within each replicate. The trials included five different solo fungicides at full dose and an untreated control; the details of the fungicide doses are shown in **Table 1**. Fungicide application was performed at growth stage 39 (Zadoks et al., 1974) using a bicycle plot sprayer at low pressure (3 bar), flat fan nozzles, and water volume of 300 l ha^-1^. The winter wheat cultivar ‘KWS Emil’ was selected for both years. Local wheat-growing practices were applied for other plant protection measures and fertilization.

**Table 1:** Fungicide treatments (active ingredients g ha^-1^) applied in the field trials at GS 39.

Treatment	MoA	g a.i. ha^-1^
Untreated	–	–
Azoxystrobin	QoI	250
Pyraclostrobin	QoI	250
Fluxapyroxad	SDHI	125
Benzovindiflupyr	SDHI	75
Prothioconazole	DMI	200

Disease assessments on three upper leaves were carried out at growth stages 39, 59, and 77according to EPPO guideline 1/26 (4) at. The severity of the disease per season was expressed by the area under the disease progress curve (AUDPC) value (Simko and Piepho, 2012):


$$AUDPC=\sum_{i=1}^{n-1}\frac{y_{i}+\;y_{i+1}}{2}x(t_{i+1}–\;t_{i})$$

where *n* is the total number of assessments; y*_i_* – disease severity (%) at the *i*th assessment; t*_i_* – days at the *i*th assessment.

The trials were harvested with a small plot harvester Haldrup C-85 (Germany). Grain yield was adjusted to 15% moisture content and converted to t/ha.

### 2.7 Statistical analysis

Statistical analysis of the experimental data was performed by applying the analysis of variance (ANOVA) using the PROC GLM procedure of software *SAS*, version 9.4 (SAS Institute, USA). Duncan’s multiple range test was selected to determine the differences between treatments (*p*< 0.05) (Raudonius, 2017).

## 3 Results

### 3.1 Fungicide sensitivity screening

Forty isolates were tested for sensitivity to prothioconazole-desthio, mefentrifluconazole, and fenpicoxamid, as 2 of the initial isolates (out of 42) were contaminated by other microorganisms. The mean EC_50_ value for prothioconazole-desthio was 0.14 mg/l, with single isolates ranging from 0.01 to 1.37 mg/l (**Table 2**). The average EC_50_ value for mefentrifluconazole was 0.28 mg/l, with single isolates ranging from 0.01 to 1.7 mg/l. The EC_50_ values of reference isolate IPO 323 was 0.01 mg/l both for prothioconazole-desthio and mefentrifluconazole. The average resistance factors for prothioconazole-desthio and mefentrifluconazole were 14 and 28, respectively. The average EC_50_ value for fenpicoxamid was 0.24 mg/l, with single isolate values ranging from 0.06 to 0.95 mg/l. The EC_50_ value of the reference isolate IPO 323 for fenpicoxamid was likewise 0.24 mg/l, therefore, the mean resistance factor for it was 1, ranging from 0 to 4.

**Table 2:** Mean EC_50_ (mg/l) values and resistance factors (RF) for prothioconazole-desthio, mefentrifluconazole and fenpicoxamid in *Z. tritici* isolates from Lithuania.

	Reference IPO323	Average	RF
Prothioconazole-desthio	0.01	0.14 (0.01-1.37)	14
Mefentrifluconazole	0.01	0.28 (0.01-1.7)	28
Fenpicoxamid	0.24	0.24 (0.06-0.95)	1

### 3.2 QoI and SDHI resistance

All 42 *Z. tritici* single pycnidium isolates were tested to determine the presence of *Cytb* gene mutation G143A which had been linked to high resistance levels to QoI fungicides. The mutation was found in 76.2% (32 out of 42) isolates (**Table 3** **).** The mutations C-T79N and C-N86S, related to resistance to SDHI fungicides, were not detected in the single pycnidium isolates.

**Table 3:** Frequency of mutations conferring resistance to QoI and SDHI fungicides in Lithuania in 2021.

Mutation	Wild type	Mutated	Frequency (%)
G143A	10	32	76.2
N86S	42	0	0
T79N	42	0	0

A total of 23 DNA samples from a bulk of leaves, originating from 23 locations throughout Lithuania, were tested to determine the frequency of the alterations C-T79N and C-N86S in SDH-C. The alteration C-T79N was not detected in these samples. The alteration C-N86S, however, was found in 3 samples at low frequencies (5.83, 8.28, and 17%).

### 3.3 DMI resistance-conferring mechanisms

**Figure 2:**
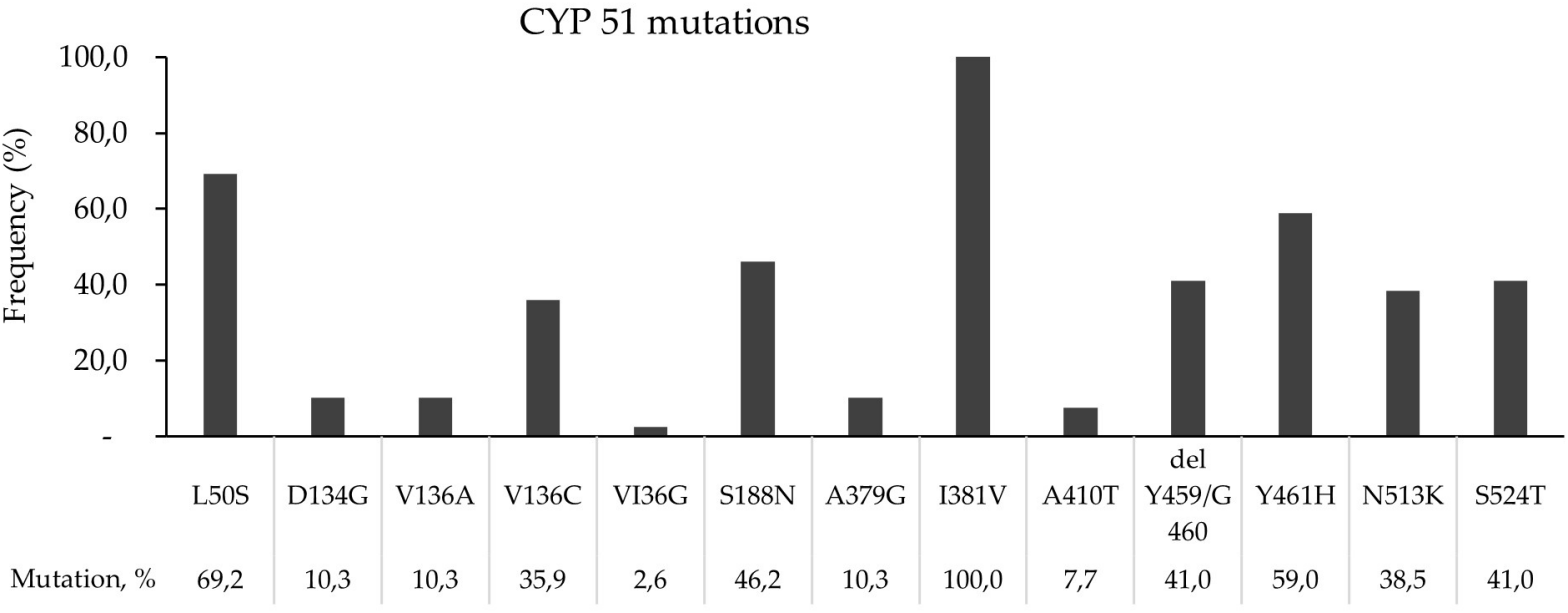
Frequency of thirteen most frequent mutations in CYP 51 gene in Lithuania 2021.

The sequencing of *Z. tritici* isolates from Lithuania revealed at least three mutations per isolate. A total of 13 target site alterations were detected amongst 39 isolates (**Figure 2**). The most frequent mutation, I381V, was found in all the isolates. Two different alterations were found at amino acid positions 459–461; either by deletion of Y459 and G460 or by the substitution Y461H. The latter was detected in 59% of the isolates, while the remaining 41% of the isolates contained the deletion of Y459 and G460. Another frequently detected mutation was L50S; this alteration was found in nearly 70% of isolates. The alteration S524T, related to decreased sensitivity to many azoles, was found in 41% of the isolates.

Across all 39 isolates of *Z. tritici*, 9 different *CYP51* haplotypes were identified (**Table 4**). Haplotype D13 showed the highest frequency, representing 30.8% of isolates. This haplotype consists of four mutations in *CYP51*: V136C, I381V, Y461H, and S524T. The second most frequent haplotype was F2 (L50S, S188N, I381V, Del459/460, and N513K), representing 28.2% of the isolates. Haplotype C8 (L50S, I381V, and Y461H) represented 10.3% of the isolates. These 3 haplotypes comprised almost 70%of the *Z. tritici* population of isolates from Lithuania. The haplotypes of the two isolates were not identified. One of those consisted of alterations L50S, S188N, A379G, I381V, A410T, Del459/460 in *CYP51*. Another one contained alterations L50S, V136G, S188N, I381V, Del459/460, N513K.

**Table 4:** The frequency of *CYP51* haplotypes of *Z. tritici* found in the isolates from Lithuania and positions of target site mutations they contain.

Haplotypes	Position of target site mutation												Frequency (%)
	L50	D134	V136	S188	A379	I381	A410	Y459	G460	Y461	N513	S524	
**C8**	S	–	–	–	–	V	–	–	–	H	–	–	**10.3**
**D13**	–	–	C	–	–	V	–	–	–	H	–	T	**30.8**
**E4**	S	G	A	–	–	V	–	–	–	H	–	–	**7.7**
**E7**	S	–	C	–	–	V	–	–	–	H	–	T	**2.6**
**F2**	S	–	–	N	–	V	–	del	del	–	K	–	**28.2**
**F4**	S	–	C	N	–	V	–	–	–	H	–	T	**5.1**
**F8**	S	G	A	–	–	V	–	–	–	H	–	T	**2.6**
**G1**	S	–	–	N	G	V	–	del	del	–	K	–	**2.6**
**H5**	S	–	–	N	G	V	T	del	del	–	K	–	**5.1**

– means no mutation at this position.

**Figure 3:**
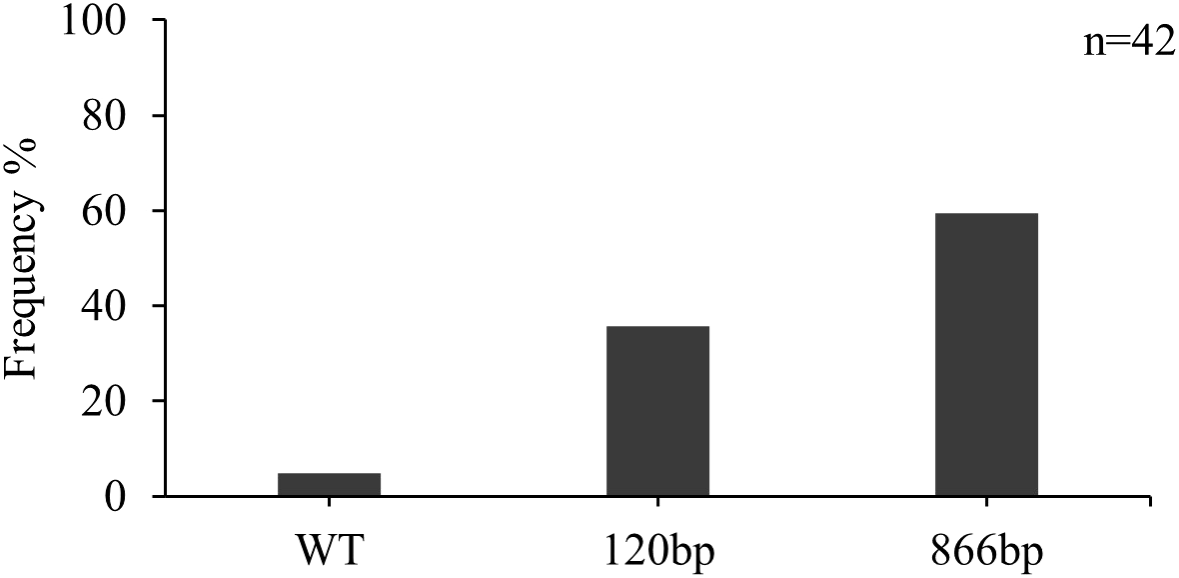
Frequency of inserts in the promoter region of the CYP51 gene (%) in *Z. tritici* isolates from Lithuania in 2021.

Forty-two isolates were screened for the presence of inserts in the *CYP51* promoter region. Two isolates from the Lithuanian population contained the wild-type promoter without inserts (**Figure 3**). The insert of 120 bp was detected in 35.7% of the isolates. In the majority (59.5%) of the isolates, the 866 bp insert was detected.

The assay to determine possible MDR revealed that one of the isolates (21-ZT-LT-14-01) had a promotor insert in the *MFS1* gene. It was determined to be a novel type of insert.

### 3.4 Field experiment

The field efficacy of fungicides with different MoA was determined *via* the area under disease progress curve (AUDPC) values. Field experiments were carried out in 2020 and 2021; average AUDPC values varied from 43.65 to 184.53 (**Table 5**). The results from field trials showed moderate control of STB in field trials, however, treatments showed significant field efficacy. Fluxapyroxad showed the highest efficacy in both years by decreasing the AUDPC by 76.2 and 63.1% in 2020 and 2021, respectively. The field efficacy of azoxystrobin was the lowest, yielding 42.0 and 38.7% in 2020 and 2021, respectively.

**Table 5:** The area under disease progress curve (AUDPC) of winter wheat infected by *Z. tritici* in 2020 and 2021.

Treatment	g ha^-1^	2020		2021	
		AUDPC	% Control	AUDPC	% Control
Untreated	–	184.53 c	–	133.26 d	–
Azoxystrobin	250	106.95 b	42.0	81.75 c	38.7
Pyraclostrobin	250	88.32 b	52.1	60.45 ab	54.6
Fluxapyroxad	125	43.65 a	76.2	49.19 a	63.1
Benzovindiflupyr	75	46.74 a	74.7	65.50 abc	50.9
Prothioconazole	200	99.91 b	45.9	76.81 bc	42.4

Different letters represent significant differences (p<0.05).

All treatments resulted in increased grain yield, improving it within the ranges of 0.1-5.1% and 2.2-14.2% in 2020 and 2021, respectively, in comparison with untreated control (**Table 6**). In both years, the highest significant grain yield increase was obtained from treatment with fluxapyroxad; the increase reflected the level of disease control. The lowest yield increase was determined by applying azoxystrobin and pyraclostrobin in 2020 and 2021, respectively.

**Table 6:** Effect of fungicides on the grain yield of winter wheat in 2020 and 2021.

Treatment	g ha^-1^	2020		2021	
		Yield t ha^-1^	Yield increase, %	Yield t ha^-1^	Yield increase, %
Untreated	–	8.89 a	–	6.93 a	–
Azoxystrobin	250	8.90 a	0.1	7.45 cd	7.5
Pyraclostrobin	250	9.05 abc	1.8	7.08 ab	2.2
Fluxapyroxad	125	9.34 c	5.1	7.92 f	14.2
Benzovindiflupyr	75	9.17 abc	3.2	7.31 bc	5.4
Prothioconazole	200	9.15 abc	2.9	7.74 def	11.6

Different letters represent significant difference (P<0.05).

## 4 Discussion

The first research project, aiming to evaluate the sensitivity levels of *Z. tritici* to DMI fungicides in Lithuania was by Ronis et al., 2014. Subsequently, studies performed by researchers from Denmark (Wieczorek et al., 2015; Heick et al., 2017) and Estonia (Mäe et al., 2020) included few samples from Lithuania in their studies. The latter screened not only the sensitivity to DMI fungicides, but also QoI and SDHI fungicides and mechanisms related to resistance occurrence (Mäe et al., 2020). Therefore, the present study was carried out to identify any possible changes and determine the current status of fungicide resistance in the Lithuanian *Z. tritici* population.

DMI fungicides have been used for almost 40 years in Lithuania, where they are essential fungicides for STB control, as in the rest of Europe. Ronis et al. (2014) reported a decline in sensitivity of the Lithuanian *Z. tritici* population to DMI fungicides epoxiconazole and cyproconazole between 2009 and 2011. A fungicide sensitivity test was carried out with two DMI fungicides: prothioconazole-desthio and mefentrifluconazole. Moreover, the frequencies of *CYP51* mutations, which are related to sensitivity decrease, were investigated. Furthermore, in this study, the isolates were classified by the number of alterations in the *CYP51* gene.

Prothioconazole has been used since 2006 in mixtures with other active ingredients. However, as a solo product it was registered only in 2018 (VATZUM, 2018). Although the EC_50_ values in this study for prothioconazole-desthio varied, the mean value remained similar to the one reported in 2020 (Mäe et al., 2020); therefore, no visible shift was observed. In the field experiment, prothioconazole had a similar control of STB as was showcased in 2014 by Ronis et al., thus reflecting an observed stabilization of DMIs in 2019 (Heick et al., 2020).

One isolate stood out due to its high EC_50_ value for prothioconazole-desthio (1.37 mg/l). This isolate contained a combination of mutations D134G, V136A, and S524T. This combination was confirmed to be related to reduced sensitivity for prothioconazole (Cools and Fraaije, 2013; Kildea et al., 2019). The isolates from this study, possessing a combination of D134G and V136A, had low EC_50_ values for prothioconazole-desthio, as opposed to the findings of Huf et al. (2018). Overall, this study showed an increase in the frequency of mutation S524T, which significantly influences DMI sensitivity (Cools et al., 2011). As seen over the past decade, there is a tendency of increasing mutation S524T and V136C frequencies in the Lithuanian Z. tritici population (Wieczorek et al., 2015; Heick et al., 2017; Mäe et al., 2020). The mutation I381V was found in all investigated isolates, thus continuing to be the dominant mutation in Lithuania (Heick et al., 2017; Mäe et al., 2020).

Mefentrifluconazole was introduced in the European and Lithuanian markets in 2020: It is a new DMI group fungicide with superior efficacy against *Z. tritici* amongst other fungicides in the group (Bryson et al., 2018; Jørgensen et al., 2020). The sensitivity test with mefentrifluconazole revealed high diversity in EC_50_ values. The reason for this variation could be linked to the previous findings showing the cross-resistance pattern. Other studies revealed a strong correlation between the sensitivity of mefentrifluconazole and tebuconazole (Mäe et al., 2020), as well as trifluconazole and difenoconazole (Cools and Fraaije, 2013). Although it is unknown if mefentrifluconazole selects for specific alterations, the isolates carrying the mutation A410T (7.7%) had higher EC_50_ values for mefentrifluconazole.

The overexpression of the target gene, which is likewise associated with reduced sensitivity towards fungicides, was screened in this study. The 866 bp insertion at higher frequencies was already found in the Lithuanian *Z. tritici* population back in 2017 (Heick et al., 2017). However, the 120 bp insertions were discovered for the first time by Mäe et al., 2020. Our findings showed an increase in the frequency of the latter type of insertion. The 120 bp insertion was primarily found in isolates with *CYP51* haplotype F2 (L50S, S188N, I381V, Del459/460, N513K), in accordance with studies carried out in other countries (Cools et al., 2012; Huf et al., 2018).

Enhanced active fungicide efflux, also known as multi-drug resistance (MDR), has also been reported as a resistance mechanism. MDR is explained as the overexpression of the “major facilitator gene” (*MFS1*). So far, 3 types of inserts (type I, II, and III) in the promoter region were identified concerning *MFS1* overexpression (Leroux and Walker, 2011; Omrane et al., 2017). In the present study, none of these were detected; however, we found one insertion that had not been reported previously. Initial studies do not implicate that this novel insert is associated with enhanced efflux activity (data not shown).

As mentioned previously, alteration of *Cytb* at location G143 is responsible for the resistance to QoI group fungicides. Our results showed that the frequency of this mutation in the Lithuanian *Z. tritici* population increased slightly in comparison to the results obtained in 2020 by A. Mäe et al. Moreover, it quadrupled over the past eight years (Heick et al., 2017), thus, explaining the relatively low efficacy of azoxystrobin and pyraclostrobin in the field trial. Fenpicoxamid is a relatively new active ingredient in fungicides, demonstrating a novel mode of action (QiI), and is currently not registered in Lithuania. Fouché et al. (2021) linked the alteration G37V in *Cytb* to reduced sensitivity in *Z. tritici* strains, therefore, future monitoring for fenpicoxamid is essential. In this study, the sensitivity test with fenpicoxamid was performed; these results can serve as baseline sensitivity data in future investigations.

Although the SDH subunit C has numerous mutations, the alterations T79N and N86S are the most frequent and had been linked to reduced sensitivity to SDHI fungicides (Rehfus et al., 2018; Hellin et al., 2021). In the present study, these mutations were not detected in *Z. tritici* single isolates. Similarly, none of these mutations were identified in Estonia, Latvia, or Poland in 2019 (Hellin et al., 2021). The field experiment further confirms these findings, as fluxapyroxad had the highest efficacy against the disease. No single isolates were found to harbour C-T79N or C-N86S, which had been associated with reduced field efficacy when present in high frequencies. However, when assessing the frequency of these mutations in bulk samples, the mutation N86S was found in 3 samples, which suggests that these mutations are starting to occur in our region.

The more frequent development of resistance to fungicides is induced by the chosen control measures (Heick et al., 2017; Jørgensen et al., 2021a). Several measures had already been investigated to delay the emergence and spread of fungicide resistance in pathogen populations, and to ensure effective disease control. Researchers suggest strategies that include choosing resistant varieties, using fungicide mixtures and dose rate changing (Hobbelen et al., 2014), minimizing the number of fungicide applications by selecting appropriate application timings (Verikaite et al., 2022), and other integrated pest management (IPM) measures (Ponomarenko et al., 2011).

## 5 Conclusions

The current fungicide resistance status in *Z. tritici* in Lithuania follows the tendencies observed in other European countries. While the field efficacy of QoI group fungicides has been significantly comprised by the advent and spread of the target site mutation G143A, a more gradually shifting field efficacy of DMI fungicides is observed. This has been associated with the complexity of *CYP51* alteration combinations and a more distinct cross-resistance pattern. Presently, no reduction in field efficacy of SDHI fungicides in Lithuania is reported, however, the first occurrences of mutations are being recorded. Resistance to the newly released DMI mefentrifluconazole and the QiI fenpicoxamid fungicides was observed at sufficiently low levels. Nevertheless, to prolong the longevity of both old and new active ingredients, the integration of anti-resistance measures embedded in an IPM approach should be imperative.

## Data availability statement

The raw data supporting the conclusions of this article will be made available by the authors, without undue reservation.

## Author contributions

KL: investigation, writing–original draft, writing–review and editing, visualization. TH: conceptualization, methodology, writing–review and editing. JR: supervision, writing–review and editing. RA: writing–review and editing. AR: writing–review and editing. All authors contributed to the article and approved the submitted version.
