# Supplementary material for: Fungicide sensitivity levels in the Lithuanian Zymoseptoria tritici population in 2021
Source: Front Plant Sci. 2023 Jan 11;13:1075038. doi: 10.3389/fpls.2022.1075038 (PMC9875010; doi:10.3389/fpls.2022.1075038)
Supplement: Supplementary file 1 [file Table_1.docx]

Table S1 primer used to analysis *CYP51* (Kildea et al. (2019))

| Primers | Sequence | Amplicon size (bp) |
| --- | --- | --- |
| C51-F1 | ACCTGCAGGCAGAACTAAGC | 1082 |
| C51-R1 | CCTCCTGTGCCTGACTTCAC |  |
| C51-F2 | TCGCGGACCTCTACCACTAC | 851 |
| C51-R2 | GTATTTCTCGGACGGGCTCT |  |
| C51-F3 | GCAAATACAAGGACGGCAAT | 944 |
| C51-R3 | GGACAGGATGTGGTCTGGAT |  |

**Figure S1 The electrophoresis gel pictures for the insertions in the *CYP51* promoter region (The isolate IPO 323 (Dutch field strain first isolated in 1984) was included as a reference).**
